# Intraoperative Neuromuscular Monitoring: Electromyography Monitor Versus Peripheral Nerve Stimulator. Does the Face Lie?

**DOI:** 10.14740/jmc5267

**Published:** 2026-03-04

**Authors:** Edison E. Villalobos, Stephania Paredes Padilla, Sorin J. Brull, Joseph D. Tobias

**Affiliations:** a Department of Anesthesiology & Pain Medicine, Nationwide Children’s Hospital, Columbus, OH, USA; b Department of Anesthesiology & Perioperative Medicine, Mayo Clinic College of Medicine & Science, Jacksonville, FL, USA; c Department of Anesthesiology & Pain Medicine, The Ohio State University College of Medicine, Columbus, OH, USA

**Keywords:** Neuromuscular block, Neuromuscular blocking agents, Train-of-four, Peripheral nerve stimulator, Quantitative neuromuscular monitor

## Abstract

During intraoperative anesthetic care, the administration of neuromuscular blocking agents (NMBAs) may be required to facilitate endotracheal intubation or provide ongoing skeletal muscle relaxation during surgical procedures. Assessments of the train-of-four (TOF) responses may be used to judge the adequacy of block and the need for redosing of NMBAs. The TOF evaluation can be performed qualitatively, by manual palpation or visual evaluation of the twitch response, or quantitatively, by using devices that measure the amplitude of the twitches. As an operator-dependent technique, qualitative techniques are prone to error, particularly in pediatric patients, and are not sensitive enough to detect a clinically significant TOF fade, which may increase the risk of postoperative residual neuromuscular block. We present a pediatric case of intraoperative neuromuscular monitoring that demonstrates discrepancies between an electromyography (EMG)-based quantitative monitor and qualitative TOF assessments using a peripheral nerve stimulator. The different sensitivities between respiratory and peripheral muscles to NMBAs, as well as the difference between qualitative and quantitative TOF monitoring are discussed.

## Introduction

In 1970, Ali et al introduced the train-of-four (TOF) as an objective method to assess neuromuscular block (NMB) by delivering a sequence of four electrical stimuli at a frequency of 2 Hz (0.5 s intervals between each stimulus) to the ulnar nerve and expressing the height of the fourth twitch as a percentage of the first, thereby providing a TOF ratio or TOFR [1]. TOF monitoring of the neuromuscular junction can be used to guide the intraoperative administration of neuromuscular blocking agents (NMBAs), helping providers determine the efficacy of the initial dose, readiness for endotracheal intubation, timing of redosing, and establishing adequate recovery of neuromuscular function before tracheal extubation [2].

Assessments of TOF responses can be performed qualitatively, by manual palpation or visual evaluation of the twitch response, or quantitatively, by using devices that measure the number and amplitude of the twitches, providing a TOF count (TOFC) and TOFR, respectively [3, 4]. Common quantitative monitors include mechanomyography (MMG), acceleromyography (AMG), and electromyography (EMG)-based devices, and current guidelines recommend the use of quantitative monitoring at the adductor pollicis muscle to assess neuromuscular function and adequacy of neuromuscular recovery [4–6]. Despite these recommendations, peripheral nerve stimulators (PNSs) for subjective (visual and tactile) TOF assessments remain a common practice among providers in the United States. As an operator-dependent technique, qualitative techniques are prone to error, particularly in pediatric patients, and may not be sensitive enough to detect clinically significant TOF fade, which increases the risk of postoperative residual NMB [5–7]. We present a pediatric case of intraoperative neuromuscular monitoring that demonstrates discrepancies between an EMG-based quantitative TOF monitor and qualitative TOF assessments using a PNS. The primary objective of this case report is to demonstrate the clinical value of EMG-based quantitative TOF monitors over a qualitative monitor (PNS), and to highlight how qualitative monitoring, particularly at the face, can mislead management decisions. We also aim to illustrate the different sensitivities between respiratory and peripheral muscles to NMBAs.

## Case Report

This review followed the guidelines of the IRB of Nationwide Children’s Hospital (Columbus, Ohio), and was conducted in compliance with the ethical standards of the responsible institution on human subjects as well as with the Helsinki Declaration.

The patient was an 8-year-old, 22.9 kg, female with extrahepatic portal vein thrombosis and cavernous transformation complicated by portal hypertension, esophageal varices, splenomegaly, and pancytopenia. Family history was notable for coagulation disturbances with thrombotic complications including factor V Leiden mutation. Past surgical history was relevant for bilateral tympanostomy tube insertion and adenoidectomy at 1 year of age. At the age of 7 years, she presented to the emergency department with complaints of flu-like symptoms, along with intermittent abdominal pain. The following night, she developed melena and hematemesis requiring the transfusion of one unit of packed red blood cells, and subsequent admission to the hospital’s gastrointestinal service. Esophagogastroduodenoscopy revealed esophageal varices requiring band ligation, and colonoscopy demonstrated rectal varices. Imaging studies revealed portal hypertension and extensive collateral vessel formation, including splenorenal collaterals, upper abdominal varices, as well as porta-hepatic and intrahepatic collaterals. She was referred to our institution for further evaluation, where additional studies confirmed extra hepatic portal vein thrombosis with cavernous transformation. Hematology workup for inherited or acquired thrombophilia was negative and she was diagnosed with anemia secondary to blood loss and pancytopenia due to splenic sequestration.

After discussing surgical options, she underwent a meso-spleno shunt and umbilical hernia repair. Although there were no immediate intraoperative complications, the patient’s trachea remained intubated and she was transferred to the pediatric intensive care unit (PICU) for postoperative care and ventilatory support. Her postoperative course was complicated by respiratory insufficiency and failure of the meso-spleno shunt due to thrombosis of the conduit, a common postoperative complication. Her trachea was extubated on postoperative day (POD) 3, and she remained in the PICU until POD 6, when she returned to the operating room (OR) for takedown of the failed meso-spleno shunt and creation of a distal spleno-renal shunt. At the time of the procedure, her medication regimen included analgesia with continuous intravenous (IV) infusion of ketamine (0.18 mg/kg/h) and hydromorphone patient-controlled analgesia (continuous 0.1 mg/h with 0.1 mg boluses every 10 min as needed), anticoagulation with heparin (10 units/kg/h), and maintenance fluids. Scheduled antibiotic therapy included cefepime and metronidazole, and additional supportive medications included diazepam, pantoprazole, phytonadione (vitamin K1), and electrolyte replacements as needed. On anesthesia pre-evaluation, the patient’s weight was 22.9 kg, and her vital signs showed a temperature of 36.6 °C, pulse 99 beats/min, blood pressure 101/63 mm Hg, respiratory rate 22 breaths/min, and oxygen saturation of 95% on room air. Cardiac, airway, and respiratory examinations were otherwise unremarkable. She was pre-medicated with IV midazolam (2 mg) and dexmedetomidine (20 µg) and transported to the OR for induction of general anesthesia.

**Figure 1:**
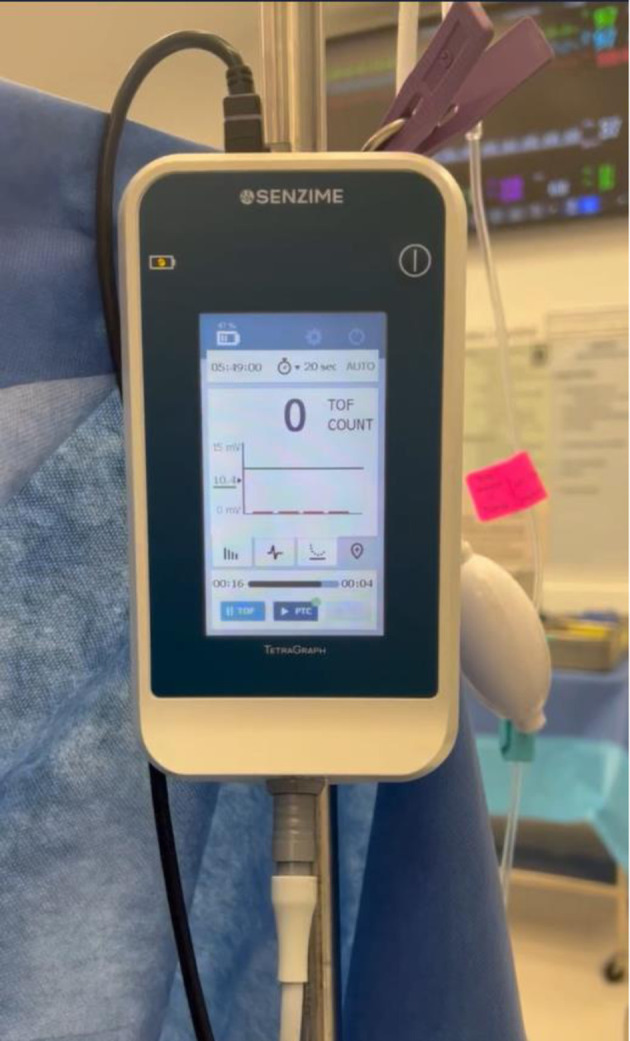
TetraGraph™ electromyography based quantitative TOF monitor (Senzime BV, Uppsala, Sweden). The displayed TOFC is 0/4. TOFC: train-of-four count.

**Figure 2:**
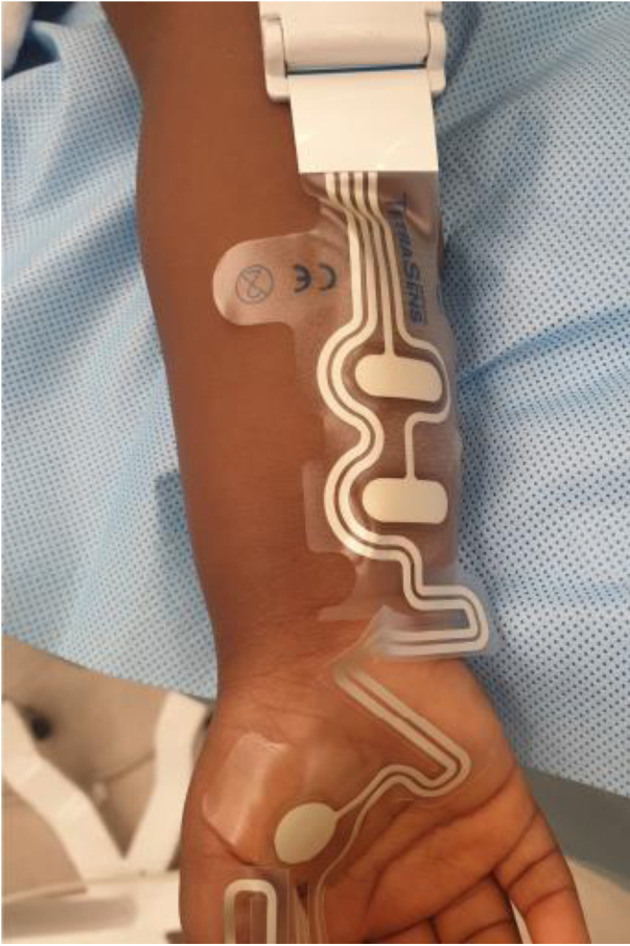
TetraSens™ (Senzime AB) pediatric size strip with recording electrodes on the palmar surface (adductor pollicis muscle) and stimulating electrodes over the ulnar nerve. This is an illustrative image of placement and is not from the reported case.

Routine American Society of Anesthesiologists’ monitors were placed, and general anesthesia was initiated with an IV dose of ketamine (2 mg/kg), followed by lidocaine (1.5 mg/kg) and methadone (0.15 mg/kg). Depth of anesthesia was titrated according to clinical signs. A depth of anesthesia monitor was not used. Neuromuscular block was achieved with an IV bolus dose of rocuronium (1.2 mg/kg) and quantitative TOF data were obtained using a TetraGraph™ EMG-based monitor (Senzime BV, Uppsala, Sweden) (Fig. 1). The TetraSens™ strip sensor was placed along the ulnar nerve on the volar aspect of the distal forearm to provide neurostimulation and the monitor was set at a 20-s interval between pulses for TOFC and TOFR (Fig. 2). Deep block assessments were obtained with post-tetanic count (PTC) measurements at the default 2-min interval of the TetraGraph™, which can be increased to 3, 5, 10, or 15 min. This preset setting for PTC does not induce post-tetanic facilitation and did not therefore affect subsequent TOF measurements [8]. Due to insufficient space on the hand secondary to existing vascular access, we were unable to initiate neuromuscular monitoring before the administration of rocuronium.

The TetraGraph™ was activated immediately after the induction of anesthesia. Monitor calibration (automatic detection of supramaximal current of 40 mA and amplitude of the single twitch of 10.44 mV) were established, and the TOFR was 86%. A subsequent TOF stimulation recorded 20 s later revealed a first twitch amplitude of 8.91 mV, with a TOFR of 93%. Additional TOF stimulations at 20 s intervals demonstrated TOFRs of 54% and 65%, respectively. Deep block (TOFC = 0) was achieved 80 s after baseline. Oral endotracheal intubation was performed without difficulty within 90–100 s following the administration of rocuronium using a 5.5 mm cuffed endotracheal tube. No patient movement was noted in response to endotracheal intubation. Once neuromuscular function recovered to 5–10 post tetanic twitches, a continuous rocuronium infusion was started at 0.6 mg/kg/h and titrated upward or downward as needed to maintain a TOFC of 1/4. If two twitches (TOFC 2/4) returned, a supplemental IV bolus of rocuronium (0.2–0.6 mg/kg) was administered, followed by an increase in the infusion rate. The specific supplemental bolus dose was selected by the anesthesia provider based on an assessment of the intraoperative surgical requirements for NMB (this protocol is standard for our current practice). Anesthesia was maintained with isoflurane (0.8–1.2%) in air/oxygen, along with a continuous infusion of ketamine (0.25–0.35 mg/kg/h, titrated based on hemodynamic and analgesic needs), dexmedetomidine (1 µ g/kg/h), and intermittent doses of fentanyl (2 µg/kg). These agents were used to improve hemodynamic stability and reduce opioid requirements based on the clinical status of the patient. Normothermia was maintained by control of the room temperature and a forced air warming blanket.

**Figure 3:**
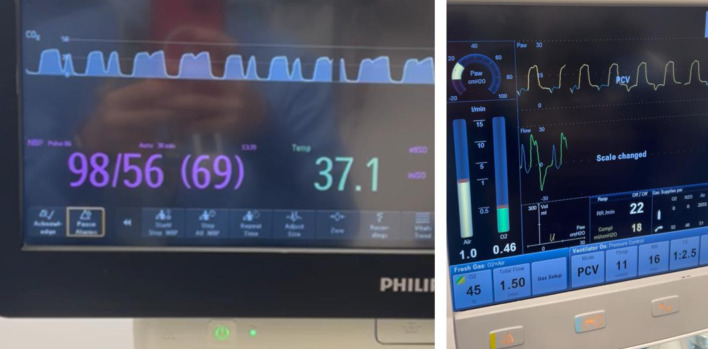
Left: end-tidal carbon dioxide (ETCO_2_) waveform showing a classical curare cleft. Right: ventilator screen displaying spontaneous respiratory efforts and ventilator settings.

Following our rocuronium dosing protocol, we were able to maintain a TOFC ≤ 1/4 as indicated by the TetraGraph™, our primary neuromuscular monitoring modality throughout the procedure. Approximately 4 h after anesthetic induction, the patient exhibited spontaneous respiratory efforts, evidenced by abnormal waveforms (“curare cleft”) on the ETCO2 capnography screen (Fig. 3). Pressure controlled ventilation (PCV) mode was maintained via the endotracheal tube and no changes were made to the ventilator settings during episodes of spontaneous respiratory efforts. Inspired and expired isoflurane was titrated to achieve the required depth of anesthesia based on clinical parameters. At that time, the TetraGraph™ displayed a TOFC of 1/4, whereas attempted visual cross validation with data from a PNS placed on the orbicularis oculi muscle showed 4/4 twitches. This temporary discrepancy between clinical signs and the TetraGraph™ prompted the decision to administer a bolus dose of rocuronium (0.6 mg/kg) and increase the infusion rate to 0.48 mg/kg/h. Toward the end of the procedure, the rocuronium infusion was stopped; however, 6 min later, the patient demonstrated respiratory efforts again. At that time, the TetraGraph™ showed TOFC = 0/4 twitches and visual cross validation with the PNS on the orbicularis oculi showed TOFC = 2/4, prompting the administration of a supplemental bolus dose of rocuronium (0.2 mg/kg). Forty-three minutes later, with the TetraGraph™ displaying a TOFC of 1/4 and visual PNS assessments on the orbicularis oculi showing 4/4 TOF twitches, NMB was reversed with a bolus dose of sugammadex (4 mg/kg). The TetraGraph™ remained the primary neuromuscular monitoring guide, including assessment of reversal of NMB at the completion of the surgical procedure. Within 90–120 s, the TOFR was ≥ 0.9; however, the patient’s trachea was extubated approximately 15 min later to ensure adequate spontaneous ventilation and safe transfer to the PICU as part of her postoperative care plan.

The patient remained hemodynamically stable throughout the procedure and there were no further intraoperative anesthetic concerns or complications. During the 7 h and 30 min of anesthetic care, the patient received 1,000 mL of IV Normosol-R, 250 mL of 5% albumin, and 309 mL of packed red blood cells. Estimated blood loss was 200 mL and total urine output was 475 mL. The patient was transferred to the inpatient ward on POD 1 and discharged home on POD 11. A timeline of rocuronium administration and neuromuscular monitoring assessments is provided in Table 1.

**Table 1:** Timeline of Rocuronium Administration and Neuromuscular Monitoring Using EMG and PNS

Absolute time	Rocuronium bolus (mg/kg)	Rocuronium infusion rate (mg/kg/h)	EMG TOFC (twitches)	PNS assessment	Event
8:05	—	—	—	—	Induction
8:17	1.2	—	Not assessed	Not assessed	
8:19	—	—	0/4	Not assessed	Endotracheal intubation
8:52	—	Start (0.6)	1/4	1/4	
9:04	0.4	0.72	2/4	2/4	
9:55	—	0.6	0/4	0/4	
10:47	—	0.48	0/4	1/4	
11:07	—	0.48	0/4	1/4	
11:40	—	0.36	0/4	2/4	
12:08	—	0.36	1/4	4/4	
12:15	—	0.36	1/4	4/4	Onset of spontaneous breathing
12:29	0.6	0.48	1/4	4/4	
12:31	—	0.48	0/4	0/4	
12:52	—	0.48	0/4	1/4	
13:52	—	0.48	0/4	2/4	
14:01	—	Stop	0/4	2/4	
14:07	—	—	0/4	2/4	Onset of spontaneous breathing
14:08	0.2	—	0/4	2/4	
14:51	—	—	1/4	4/4	Sugammadex administration
15:08	—	—	4/4 (TOFR was ≥ 0.9)	Not assessed	Tracheal extubation

EMG: electromyography; PNS: peripheral nerve stimulator; TOFC: train-of-four count; TOFR: train-of-four ratio.

## Discussion

Our case illustrates challenges that may be encountered when using different methods to monitor intraoperative NMB. An understanding of these different techniques and the varying sensitivities of various muscular beds can be used to assess the depth of NMB, the need for redosing of NMBAs, and to ensure adequate recovery. Using supramaximal single-twitch stimulation of the phrenic and ulnar nerves, Cantineau et al demonstrated that the diaphragm is more resistant to rocuronium than the adductor pollicis [9]. The effective dose required to achieve a 95% reduction of twitch height (ED95) was twice as high at the diaphragm (0.50 ± 0.20 mg/kg) when compared with the adductor pollicis (0.24 ± 0.04 mg/kg, P < 0.01). The time from the initial administration to NMB (peak effect) was longer at the diaphragm (120 ± 62s) than the adductor pollicis (80 ± 20s), whereas the diaphragm recovered sooner, showing recovery times of 8 ± 4 min vs. 17 ± 9 min (P < 0.01). These findings are consistent with previous studies describing a similar dose-response relationship of non-depolarizing NMBA potency at the diaphragm and laryngeal muscles, both of which exhibit very similar onset, offset, and peak effect [10–12]. As such, the onset and peak effect of NMBAs should be interpreted with caution. Although the onset of action of NMBAs at the diaphragm and laryngeal muscles is faster than at the adductor pollicis muscle, the peak effect is achieved more slowly. This apparent discrepancy may be explained by greater blood flow to centrally located muscles, allowing earlier drug delivery with a more rapid initial effect, as well as the high density of postsynaptic acetylcholine receptors at the neuromuscular junction of the diaphragm [13]. The onset time and peak effect of NMBAs can be further influenced by factors affecting their pharmacokinetics, including coexistent hepatic or renal disease, which should be considered during clinical management [14]. Notably, our patient demonstrated respiratory efforts even at time points when the TetraGraph™ at the ulnar nerve showed 0/4 TOF twitches. This is not surprising, as the diaphragm requires a much deeper level of block to achieve full paralysis, usually at PTC ≤ 1 [14].

Different methods can be used to monitor the level of NMB, which can be broadly divided into subjective (qualitative) and objective (quantitative) techniques. Qualitative methods include PNS, and quantitative methods include EMG, AMG, MMG, and kinemyography (KMG) [2]. The use of PNS was first described in the 1950s, arising in response to the need to determine whether the failure of full muscular recovery was due to a central cause affecting the brain or to prolonged effect of NMBAs [15]. PNSs, as the name implies, are devices that can be used to deliver controlled stimulation patterns to peripheral nerves but require subjective assessment of the degree of block by visual observation or palpation of the patient’s response to the stimuli. When initially introduced, the useful information along with the relative low cost of the devices, increased their popularity and clinical availability [16]. However, despite the widespread availability of PNS, neuromuscular monitoring is not performed routinely in many operating room settings, resulting in residual NMB and its associated morbidity [7, 8]. Subjective assessments of the depth and recovery of NMB have proven to be unreliable; in fact, the use of PNS alone and clinical tests such as the 5-s head lift, grip strength, or tidal volume, are discouraged. Current guidelines strongly recommend objective (quantitative) neuromuscular monitoring instead [5, 6]. Quantitative monitors measure the strength of muscle contraction (AMG, MMG, KMG) or electrical activity (EMG), and quantify the degree of NMB, displaying numerical results on a screen (usually from 0% to 100%) [17]. AMG measures acceleration of a stimulated muscle, MMG measures isometric force of contraction, KMG measures electrical signals generated by deformation of a sensor strip, and EMG measures compound muscle action potentials following nerve stimulation. In our case, we used the TetraGraph™ EMG-based monitor (Fig. 1).

EMG monitors offer several advantages over AMG, MMG, or KMG. Quantitative EMG monitoring is not affected by changes in muscle contractility, is less dependent on normothermia, and does not require the target muscle to move freely because it measures electrical response (compound action potentials) instead of mechanical response (muscle movement). This facilitates reliable NMB monitoring even when access to the extremities is limited or the arms are tucked. EMG electrodes can also be placed on various muscle groups, including hand muscles such as adductor pollicis, first dorsal interosseous, and abductor digiti minimi [8, 17]. When neither hand is available, the foot has been described as an alternative option as well (flexor hallucis brevis muscle) [18].

The most common anatomical sites for neuromuscular monitoring are the ulnar nerve, with the evoked response in the adductor pollicis muscle (thumb adduction), and the facial nerve, with the evoked response in the orbicularis oculi or corrugator supercilii muscles (eye closure and eyebrow contraction, respectively) [17]. Therefore, it is important to highlight that the sensitivity of various muscles to NMBAs is widely variable and muscle-dependent. While the diaphragm exhibits the lowest sensitivity (it is most resistant to total block), the pharyngeal muscles demonstrate the greatest sensitivity (they are blocked first and recover last). Evoked response to a particular monitored muscle may not reflect recovery of other muscles [14, 17]. Despite their proximity, the orbicularis oculi and corrugator supercilii differ significantly in their responses to NMBAs.

Patients having qualitative monitoring at the facial nerve had a fivefold higher risk of postoperative residual NMB compared with those monitored at the adductor pollicis [19, 20]. This is due to significant differences in the degree of NMB sensitivity between peri-ocular muscles and the adductor pollicis muscle. Additionally, clinicians may be unable to distinguish evoked twitches from different muscles surrounding the eye versus direct stimulation of the facial muscles (which bypasses the neuromuscular junction). Evidence suggests that the corrugator supercilii, rather than the orbicularis oculi, is primarily responsible for this observed resistance when monitoring “eye muscles”, leading clinicians to count corrugator supercilii twitches (more resistant), even when the orbicularis oculi is fully blocked (less resistant). In fact, the corrugator supercilii muscle correlates better with onset and degree of NMB at the laryngeal muscles. Another limitation is that surrounding muscles of the face may be activated by facial nerve stimulation or direct muscle stimulation, further impeding clinicians’ assessments [21].

Beyond operator-dependent factors, different sensitivities to NBMAs can be explained by anatomic and physiologic characteristics. For example, orbicularis oculi fibers are small, rounded, and composed of approximately 89% fast-twitch type-II fibers, whereas corrugator supercilii fibers are larger, polygonal, and composed of approximately 49% fast-twitch type II fibers. Moreover, the capillary area per contractile unit of the corrugator supercilii is also about 2.4 times that of the orbicularis oculi [22]. For all these factors, neuromuscular monitoring at the face, particularly at the corrugator supercilii, may suggest recovery while the adductor pollicis remains partially paralyzed, which may prompt early redosing of NMBAs and underestimation of residual NMB at the time of reversal. Indeed, as noted in our case, PNS at the facial nerve demonstrated that the “eye muscles” recovered earlier than the adductor pollicis across multiple time points (Table 1). Our case demonstrates how a temporary discrepancy between EMG-based TOF monitoring and PNS assessments can mislead NMB management decisions.

Assessment of evoked responses from the orbicularis oculi and corrugator supercilii muscles is not only difficult but is not recommended by the ASA and European neuromuscular monitoring guidelines [5, 6]. Given the variable onset times, recovery, and sensitivities across muscle groups, residual NMB has been defined as a TOFR < 0.9 specifically at the adductor pollicis muscle. The adductor pollicis muscle is preferred because it is easily accessible intraoperatively, the risk of direct muscle stimulation is minimal, it recovers more slowly than muscles surrounding the eye and later than critical muscles such as the diaphragm and larynx. These characteristics make the ulnar nerve with evoked response measured at the adductor pollicis muscle an optimal site to assess NMB recovery, guide dosing of reversal agents, and determine readiness for tracheal extubation, ultimately minimizing the risk of residual NMB. It is acknowledged that the adductor pollicis muscle may not always be accessible intraoperatively, and in such cases monitoring at the “eye muscles” is sometimes performed. However, in this scenario, guidelines recommend switching monitoring to the adductor pollicis before reversal, as dosing of reversal agents is based on adductor pollicis responses (and depth of block). Despite these recommendations, adoption of this practice has not been universal, especially when caring for pediatric patients. Importantly, these guidelines are based on adult data and pediatric-aged patients present additional challenges. In children, responses to both NMBAs and antagonists such as sugammadex are more variable, patient size limits provider reach, space on the volar aspect of the arm may be insufficient for adult sensor placement, and clinical exposure to newer EMG-based monitors such as the TetraGraph™ remains limited [23]. Since European pediatric guidelines for the management of neuromuscular block have recently been announced, these limitations highlight the need for pediatric specific guidelines in the United States.

In summary, we present a pediatric case of intraoperative neuromuscular monitoring discrepancies using the TetraGraph™ EMG-based TOF monitor and cross-validation with a PNS in an 8-year-old female undergoing takedown of a failed meso-spleno shunt and creation of a distal spleno-renal shunt. Our case illustrates the different sensitivities of respiratory and peripheral muscles to NMBAs, as well as the clinically relevant difference between subjective evaluation and quantitative monitoring of NMB. Additionally, we highlight the proximity of the facial nerve to the corrugator supercilii and orbicularis oculi muscles which warrants caution, as direct muscle stimulation and difficulties distinguishing evoked responses are particularly likely when stimulating the facial nerve. These limitations are addressed in the recent ASA guidelines, which specifically discourage both the use of the face as a site for neuromuscular monitoring and subjective PNS evaluation.

### Learning points

Our case demonstrates that monitoring at the face did not provide a reliable estimation of the depth and recovery of NMB. Each muscle behaves differently in response to NMBAs because each muscle exhibits different anatomic and physiologic profiles, underscoring the large variability in muscular resistance or sensitivity to NMBAs. We acknowledge that this is a single-patient case report and that our findings may not be generalizable to all pediatric patients. Still, subjective assessments are unreliable and discouraged, and quantitative TOF monitoring at the ulnar nerve with evoked response at the adductor pollicis muscle has become the gold standard to assess both the depth and recovery of NMB [5]. Importantly, the definition of residual NMB (TOFR < 0.9) and the dosing of reversal agents are based on adductor pollicis muscle responses (and depth of block); therefore, other monitoring sites should be avoided when possible, and when used, interpreted with caution until a direct comparison of the various muscles becomes available. Access to quantitative TOF monitors in the OR, surgical positioning, access to and stability of the monitored site, patient size, and insufficient space for sensor placement (in newborn patients) are some of the important limitations to their use, especially in pediatric-aged patients. Our case reinforces the need to implement current guidelines into daily practice and highlights the importance of further studies aimed to developing pediatric-specific guidelines or recommendations for neuromuscular block monitoring.

## Data Availability

Any inquiries regarding supporting data availability of this study should be directed to the corresponding author.

## Abbreviations

AMG: acceleromyography
EMG: electromyography
KMG: kinemyography
MMG: mechanomyography
NMBA: neuromuscular blocking agent
NMB: neuromuscular blockade
OR: operating room
PTC: post-tetanic count
TOF: train-of-four
TOFC: train-of-four count
TOFR: train-of-four ratio
